# Exposure to occupational hazards for pregnancy and sick leave in pregnant workers: a cross-sectional study

**DOI:** 10.1186/s40557-017-0170-3

**Published:** 2017-05-15

**Authors:** Jean-Bernard Henrotin, Monique Vaissière, Maryline Etaix, Mathieu Dziurla, Stéphane Malard, Dominique Lafon

**Affiliations:** 1 Department of Occupational Epidemiology, National Research and Safety Institute, 1 rue du Morvan, CS 60027, Vandoeuvre-les-Nancy, 54519 Cedex France; 2 Occupational Health Service, Santé Travail Béziers Coeur d’Hérault, Béziers, France; 3 Occupational Health Service, Santé Travail Loire Nord, Roanne, France; 4 Department of Medical Studies and Assistance, National Research and Safety Institute, Paris, France; 5 Occupational Disease Consultation Centre, Raymond Poincaré Hospital, Garches, France

## Abstract

**Background:**

This study aimed to investigate the association between exposure to occupational hazards for pregnancy and sick leave (SL) in pregnant workers.

**Methods:**

A cross-sectional study was performed in French occupational health services in 2014. Occupational hazards for pregnancy were assessed by occupational health physicians (OHPs). After delivery and at the time of returning to work, 1,495 eligible workers were interviewed by OHPs. Information on SL was self-reported. Risk ratios (RRs) were calculated from multivariable analyses based on a generalized linear model with a Bernoulli distribution and a log link adjusted for selected confounders for binary outcomes or zero-inflated negative binomial regression for count outcomes.

**Results:**

Among recruited workers, 74.9% presented “at least one SL” during pregnancy. After adjustment, the cumulative index of occupational hazards (0, 1–2, 3–4, ≥ 5 risks) for pregnancy was significantly associated with “at least one SL” during pregnancy in a dose–response relationship. This gradient was also observed with “early SL” (<15 week gestation): from 1 to 2 risks, RR = 1.48 (95% confidence intervals (CIs): 0.92-2.38); from 3 to 4 risks, RR = 2.03 (95% CI: 1.25-3.30); equal to or higher than five risks, RR = 2.90 (95% CI: 1.89-4.44); with “duration of absence” (adjusted mean): from 1 to 2 risks, m = 38.6 days; from 3 to 4 risks, m = 46.8 days; equal to or higher than five risks, m = 53.8 days. We also found that deprivation, pregnancy at risk, assisted reproductive therapy, work-family conflicts, home-work commuting felt as difficult and young age are associated with a higher risk of SL.

**Conclusions:**

Our results support the assertion that pregnant workers exposed to occupational hazards for pregnancy without medical complications are also at risk of taking SL during pregnancy. More prevention in the workplace for pregnant workers exposed to occupational hazards could reduce SL.

## Background

In several European countries concerning pregnant workers, it exists in the workplace protective legislation where employers should adjust work conditions according to the needs of pregnancy [1–3]. Despite this, sick leave (SL) levels among pregnant workers have increased over the last few decades and remain extremely high today without clear explanations [4–12]. In France, pregnant workers may take legal prenatal leaves (from 6 to 8 weeks) before the date of delivery or “pathological leave” (2 weeks) in case of medical complications during pregnancy. Outside of these periods, a pregnant worker may take ordinary SL but will receive 50% of her salary. Several factors have been reported in the literature to be associated with SL during pregnancy: occupational groups [4, 8, 9, 12–14], assisted reproductive therapy (ART) [15], body mass index [14, 15], social benefits [16, 17], attitudes regarding SL in the workplace [6, 10, 16], and young women [8]. Also, some occupational exposures have also been associated with SL during pregnancy [4, 7, 13, 15, 16, 18, 19].

However, particularly in pregnant workers, studies remain scarce and occupational explanations of the high rate of SL need to be confirmed. The studies are rather small in size [7, 13], limited to certain occupations [7], without accurate exposure measurements [4, 7] and often limited or no adjustment for potential confounders [4, 7, 13]. Almost studies from the available literature have been carried out in north European countries [4, 7, 15, 17, 20–22] and to our knowledge, none in France. It is important to note that several studies report that job adjustment is associated with reduced SL during pregnancy [23, 24]. Therefore, in order to propose preventive measures in the workplace and reduce the risk of SL, it is necessary to deepen relationship between occupational exposures and SL during pregnancy.

We hypothesized that pregnant workers without medical complications during pregnancy exposed to occupational hazards for pregnancy are at risk of taking SL during pregnancy. The aim of this study was to investigate the association between exposure to occupational hazards for pregnancy and SL in pregnant workers.

## Methods

### Design and study population

A cross-sectional study was performed from January 1, 2014 to December 31, 2014 in the occupational health services of the Languedoc-Roussillon region and in the town of Roanne in France. Eighty-three occupational health physicians (OHP) participated in the recruitment of workers.

According to French labor law, each woman having worked during pregnancy must benefit from a medical visit with an OHP after delivery and at the time of returning to work. This is a compulsory visit for employers and employees. Also, at the first postnatal visit, all women (no selection method) were invited to participate in the study by the OHP. The inclusion criteria were that they: (i) must have worked for an employer during their last pregnancy (our study did not include workers without employment contract (such as self-employed, craftsman, farmer, company head…) because these workers were not followed by French occupational health services); (ii) to be older than 18 years of age (legal majority in France) (iii) must have had a postnatal visit with an OHP in the year after delivery; or (iv) within three years after delivery if they had full-time parental leave; and (v) be sufficiently fluent in French to participate in the interview. Before the medical visit, the OHPs asked eligible workers to fill-in a self-administered questionnaire to obtain sociodemographic information; during the visit, a second occupational questionnaire was administered face-to-face by the OHP. The computerized medical record system in each occupational health service was declared to the French National Commission for Data Protection. All the volunteer participants gave their informed consent to be enrolled and data were collected anonymously. Because we used anonymised data from routine medical visits, in 2013 according to French law, ethics approval was not required.

### Outcomes of interest

Information on SL during pregnancy was obtained from workers with the following questions by taking account of leaves for legal reasons and sickness: (i) for the first and second trimester of the pregnancy (“did you have one SL before a pathological or legal leave (regardless of the cause and duration)?, yes/no); (ii) on the duration (“what was the total duration of your SL, before a pathological or legal leave?, in days during pregnancy); (iii) “did you take a SL without returning to work before a pathological or legal leave ?, yes/no ? If yes, at what time of your pregnancy did you stop working completely? in which week of gestation (WG)”. In this study, we defined each trimester in the following way: first trimester when < 15 WG; second trimester from 15 WG to 28 WG. Early SL was defined as leaving job before 15 WG.

### Exposure of interest

The exposure assessment of potential hazards for pregnancy was conducted by OHPs based on knowledge of workstations in early pregnancy. Seventeen potential hazards were selected [3]: biological hazards (working with very young children, sick persons, animals); chemical hazards; night work (between 9:00.p.m and 5:00 a.m.); physical hazards (standing > 1 h a day, stair climbing (several times a day), forward bending ≥ 1 h a day, difficult postures (upper and/or lower limbs), heavy lifting > 5 kg, repetitive tasks, vibration (driving), temperature (>30 °C, <10 °C), noise >80 dB, work on industrial machines); ionizing radiation and electromagnetic fields. The responses were based on a 4-point Li﻿kert scale ranging from 1: no; 2: very rarely (a few per month); 3: sometimes (a few times a week); 4: frequently (a few times a day or more). Then, all these variables were transformed into binary variables and were coded as either 0 (to indicate the reference category) or 1 (to indicate the category at risk). For all the variables, the category at risk was the “frequently” category (level 4) except for three variables: ionizing radiation (level from 2 to 4); night work (at least one night); Electromagnetic fields (level from 3 to 4). A cumulative index of occupational hazards for pregnancy in four classes (0, 1–2, 2–4, ≥ 5 risks) was built using these seventeen occupational variables.

### Potential confounders

The choice of potential confounders was based on the literature data (age, deprivation, occupational data, number of children, assisted reproductive therapy (ART), and pregnancy at risk) except for smoking, alcohol consumption, and body mass index. We have considered that socioeconomic deprivation was a proxy measure for these last three variables because very positively correlated to them [25]. Socioeconomic deprivation was assessed using the Evaluation of Deprivation and Inequalities in Health Examination (EPICES) individual scale [25]. This is a reliable proxy in workplace settings for population-based measures of deprivation which is strongly correlated with the Townsend and Carstairs indices [25]. Workers with an EPICES score equal to or higher than 30 were classified as being deprived. Occupational skill level was classified according to the French standard classification of occupations (version 2003) from the French National Institute of Statistics [26]. The occupations were classified into four skill levels: managers/supervisors, intermediate occupations, employees, and manual workers. Women were asked if they had been followed-up for “pregnancy at risk of medical complications” (yes/no) and whether they had had assisted reproductive therapy (yes/no). Other explored factors were maternal age at delivery, company size at four levels (<10, 10–49, 50–199, ≥ 200 workers), type of contract (non-fixed term, fixed term), home-work commuting (duration, mode), working time (full-time, part-time), and job duration (<2, ≥ 2 years).

Several factors, no used in literature like confounders until now, related to concept “work-family conflict” were also used [27, 28]. This concept focuses on the difficulties that employees have in balancing their work and family responsibilities which could increase fatigue during pregnancy and the occurrence of SL [27, 28]. In our study, several predictors of work-family conflict were used as follows. An index of cumulated work-family conflict risks in four classes (0, 1, 2, ≥ 3 risks) was built using five dichotomous variables: preschool-age children at home (yes/no), home-work commuting > 50 min/d (yes/no), duration of working hours > 8 h/d (yes/no), irregular working hours (yes/no), and absence of two consecutive rest days in a week (yes/no).

### Statistical analyses

In bivariate analyses, chi-square tests were used to compare binary variables. Also for continuous variables, the Kolmogorov-Smirnov test was used to evaluate the normal distribution. A Student’s *t*-test was used to analyze the normally distributed quantitative values, and the Mann–Whitney *U* test or the Kruskal-Wallis test was used to analyze the non-normally distributed ones. In multivariate analyses for binary outcomes, adjusted relative risks (RRa) with 95% confidence intervals (CIs) were calculated based on a generalized linear model with a Bernoulli distribution and a log link adjusted for selected confounders [29]. A stepwise forward procedure was conducted to identify the variables having a significant association with the outcome. For “at least one sick leave” variable, we have conducted analyses separately for each trimester of pregnancy because the impact of the factor may be different according to the pregnancy period. To compare the results between trimesters, we have presented the significant variables from final models of the stepwise forward procedure but also the non-significant variables adjusted for these significant variables. Also, we tested interaction terms between several variables. Notably, to answer our hypothesis, analyses between “number of occupational risks for pregnancy” and “pregnancy at risk” were carried out separately when a positive interaction was identified between these two variables. For count outcome, zero-inflated negative binomial regression (ZINB) was carried out to take into account over-dispersion and/or excess of the zero value in data [30]. For this outcome the purpose of the analysis was exploratory, we have presented the results without a selection procedure of the variables. A two-sided p-value of less than 0.05 was considered statistically significant in our study. Statistical power was estimated on the basis of a binomial test with unequal group sizes (ratio 1/4). In this case to detect an effect size of RR = 2 with 80% power at a significance level of 0.05 and a control-group proportion at 5%, 1,245 pregnant workers were required. As the responses were relatively complete, analyses excluded missing data. All the statistical analyses were performed using STATA statistical software, version 14.0 (Stata Corp, College Station, Texas, USA).

## Results

**Fig. 1:**
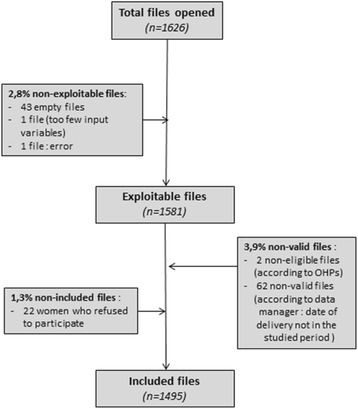
Flow chart of the study population

During the study time period, the number of workers recruited was 1,581 (Fig. 1). The number of cases excluded was 64 workers (non-valid files). Very few women refused to participate, with only 22 cases not included in the study. Thus, our final sample was composed of 1,495 workers. The mean of the proportion of missing data was 1.8% per variable with two variables above 5%: deprivation (6.2%) and job duration (6.5%).

Table 1 presents a description of the pregnant workers according to the occurrence of “at least one SL” before a pathological or legal prenatal leaves (Table 1). The proportion of “at least one SL” was 74.9%. SLs were less frequent in the managers/supervisors group and in small companies (<50 workers). Workers with “non-fixed contracts”, “pregnancy at risk” or ART had an increased proportion of SL. Note that the age mean was not different between the two groups.

**Table 1:** Characteristics of the pregnant workers according to the variable “at least one sick leave” (SL) during pregnancy

	SL				no SL				*p*-value
	(*N* = 1113)				(*N* = 373)				
	N	%	Mean	sd	N	%	Mean	sd	
Age (y)			30.6	4.5			31.1	4.4	0.08
Age group									
< 25 y	108	9.8			27	7.4			0.18
25- < 30 y	408	37.1			122	33.3			
30- < 35 y	405	36.9			151	41.3			
≥ 35 y	178	16.2			66	18.0			
Family structure									
Living with a partner (yes)	1063	96.5			352	95.4			0.31
Children at home									
0 child	577	52.1			196	52.8			0.91
1 child	449	40.5			146	39.4			
≥ 2 children	82	7.4			29	7.8			
Home-work commuting									
> 50 min/d (yes)	248	23.2			77	21.3			0.45
In car *(*yes)	1022	92.7			348	94.0			0.38
Home-work commuting felt as difficult									
Yes	245	22.3			48	13.0			<0.001
Deprivation									
EPICES score ≥ 30 (yes)	231	22.0			61	17.7			0.08
Occupational groups									
Managers/supervisors	93	8.4			51	13.7			0.001
Intermediate occupations	374	33.6			91	24.4			
Employees	591	53.1			216	57.9			
Manual workers	55	4.9			15	4.0			
Type of contract									
Non-fixed term (yes)	1508	96.1			342	92.9			0.01
Working time									
Full-time (yes)	868	78.8			300	81.3			0.31
Job duration									
< 2 years (yes)	239	21.6			90	24.4			0.27
Company size									
< 10 workers	253	23.0			114	31.2			<0.001
10 - 49 workers	332	30.2			137	37.4			
≥ 50 -199 workers	295	26.8			83	22.7			
≥ 200 workers	219	19.9			32	8.7			
Pregnancy at risk									
Yes	334	30.3			34	9.2			<0.001
Assisted reproductive therapy									
Yes	84	7.6			13	3.5			0.006

*N* number, *y* years, min *minutes, d* day, *sd* standard deviation

**Fig. 2:**
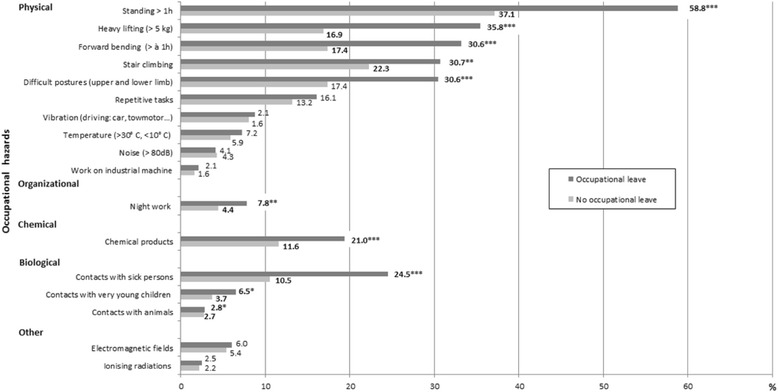
Comparison of the frequencies of occupational hazards for pregnancy according to the occurrence of “at least one sick leave” (*p < 0.05, **p < 0.01, ***p < 0.001)

Figure 2 displays the frequencies of the potential occupational hazards for pregnancy in worker groups with and without SL (Fig. 2). We observed that exposures to physical hazards (standing > 1 h, heavy lifting, forward bending, stair climbing, and difficult postures) and, although less frequent, biological hazards, exposure to chemical products and night work were significantly associated with SL.

Table 2 shows adjusted relative risks (RRa) with 95% confidence intervals for “at least one SL” from 1st trimester to 3rd trimester of gestation (Table 2). In the first trimester, the “number of occupational hazards”, deprivation, “pregnancy at risk” and ART were associated with higher RRa of SL compared to the reference group. Note that the RRa of the “number of occupational hazards” increased in a dose-dependent way. In the two other trimesters the results were presented separately for pregnant workers without and with “pregnancy at risk” because of statistical interaction between “pregnancy at risk” and the “number of occupational hazards”. Like ART variable was very correlated to “pregnancy at risk“(data not shown), this variable was not presented to second and third trimester. The gradient with “number of occupational hazards” was also observed for all the strates except for 3rd trimester in the strate with “pregnancy at risk” and the strength of effect was strongest to 2nd trimester. There were positive associations with “number of work-family conflicts” for 2nd trimester but not for 3rd trimester. The others variables (occupational group, type of contract (fixed-term), company size, home-work commuting and, age) were positively associated with outcomes but only in the strate without PR.

**Table 2:** Bernoulli generalized linear model with « at least one sick leave» (SL) as dependent variable according to 1st and 2nd trimester

	1st trimester			2nd trimester						3rd trimester					
	with & without PR (*N* = 1257)			strate without PR (*N* = 939)^a^			strate with PR (*N* = 306)^a^			strate without PR (*N* = 939)^a^			strate with PR (*N* = 306)^a^		
	RRa^b^	95% CI	*p*	RRa^c^	95% CI	*p*	RRa^d^	95% CI	*p*	RRa^h^	95% CI	*p*	RRa^i^	95% CI	*p*
Number of occupational risks^e^															
No risk	Ref		*<0.001*	Ref		*<0.001*	Ref		*<0.001*	Ref		*<0.001*	Ref		*0.25*
1 or 2 risks	1.10	0.78 - 1.54		1.96	1.44 - 2.66		1.16	0.89 - 1.52		1.32	1.12 - 1.54		1.12	0.95 - 1.32	
3 or 4 risks	1.50	1.06 - 2.12		2.05	1.48 - 2.86		1.41	1.09 - 1.82		1.55	1.32 - 1.82		1.15	0.98 - 1.35	
≥ 5 risks	1.84	1.40 - 2.50		2.46	1.81 - 3.34		1.67	1.34 - 2.08		1.57	1.35 - 1.83		1.16	1.00 - 1.34	
Work-family conflict^f^															
No risk	Ref		*0.57*	Ref		*<0.001*	Ref		*0.07*	Ref		*0.36*	Ref		*0.50*
1 risks	0.93	0.68 - 1.27		1.27	0.98 - 1.61		1.13	0.89 - 1.44		1.11	0.97 - 1.26		1.07	0.92 - 1.24	
2 risks	1.00	0.73 - 1.38		1.40	1.07 - 1.81		1.29	1.02 - 1.64		1.09	0.95 - 1.25		1.09	0.94 - 1.27	
≥ 3 risks	1.17	0.81 - 1.67		1.69	1.29 - 2.22		1.14	0.87 - 1.50		1.03	0.87 - 1.22		1.12	0.96 - 1.31	
Deprivation (EPICES score ≥ 30)															
No	Ref		*0.02*	Ref		*0.98*	Ref		*0.17*	Ref		*0.56*	Ref		*0.64*
Yes	1.28	1.04 - 1.60		1.00	0.82 - 1.22		1.09	0.96 - 1.23		0.97	0.87 - 1.08		1.02	0.93 - 1.13	
Home-work commuting felt as difficult															
No	Ref		*0.27*	Ref		*0.16*	Ref		*0.78*	Ref		*<0.001*	Ref		*0.63*
Yes	1.13	0.99 - 1.60		1.13	0.94 - 1.35		1.01	0.89 - 1.17		1.25	1.14 - 1.37		0.97	0.87 - 1.08	
Age group (years)															
< 25	1.01	0.71 - 1.43	*0.65*	1.52	1.24 - 1.85	*<0.001*	1.00	0.81 - 1.25	*0.55*	1.34	1.17 - 1.54	*<0.001*	0.89	0.70 - 1.13	*0.30*
25- < 30	0.94	0.74 - 1.20		1.04	0.87 - 1.24		1.01	0.87 - 1.18		1.14	1.04 - 1.26		1.04	0.93 - 1.16	
30- < 35	Ref			Ref			Ref			Ref			Ref		
≥ 35	0.83	0.61 - 1.23		0.97	0.73 - 1.28		0.87	0.71 - 1.07		1.02	0.88 - 1.17		0.94	0.82 - 1.08	
Occupational group															
Managers/supervisors	Ref		*0.34*	Ref		*0.001*	Ref		*0.33*	Ref		*0.006*	Ref		*0.97*
Intermediate occupations	1.11	0.70 - 1.74		1.80	1.19 - 2.70		1.36	0.90 - 1.96		1.30	1.04 - 1.60		1.00	0.81 - 1.25	
Employees	1.16	0.75 - 1.79		1.43	0.96 - 2.16^g^		1.31	0.87 - 1.96^g^		1.16	0.93 - 1.44^g^		1.02	0.85 - 1.22^g^	
Manual workers	1.54	0.89 - 2.65					-	-							
Company size (workers)															
< 10	0.76	0.52 - 1.13	*0.55*	0.85	0.69 - 1.06	*0.38*	0.88	0.72 - 1.08	*0.55*	0.79	0.69 - 0.90	*<0.001*	0.89	0.78 - 1.02	*0.40*
10 - 49	0.82	0.58 - 1.15		0.84	0.67 - 1.04		0.95	0.82 - 1.11		0.82	0.74 - 0.91		0.95	0.84 - 1.07	
50 - 199	0.82	0.58 - 1.17		0.86	0.68 - 1.09		0.91	0.77 - 1.08		0.87	0.80 - 0.96		0.94	0.82 - 1.07	
≥ 200	Ref			Ref			Ref			Ref			Ref		
Type of contract															
Non-fixed term	Ref		*0.20*	Ref		*0.008*	Ref		*0.56*	Ref		*0.007*	Ref		*0.57*
Fixed-term	1.51	0.80 - 2.85		2.06	1.21 - 3.51		1.10	0.80 - 1.50		1.47	1.11 - 1.94		1.10	0.79 - 1.51	
Pregnancy at risk															
No	Ref	-	*<0.001*												
Yes	2.12	1.72 - 2.63													
Assisted reproductive therapy															
No	Ref	-	*0.001*												
Yes	1.54	1.19 - 2.00													

*RRa* ajusted relative risk, *95% CI* 95% confidence intervals, *p* p-value, *PR* pregnancy at risk, *Ref* reference

^a^Positive interaction between variable pregnancy at risk and variable number of occupational risks at 2nd trimester and at 3rd trimester: presentation according to strate with pregnancy at risk and without pregnancy at risk

^b^Ajusted for: number of occupational risks (0, 1 or 2, 3 or 4, ≥5 risks), deprivation EPICES score ≥ 30 (yes/no), pregnancy at risk (yes/no), assisted reproductive therapy (yes/no)

^c^Ajusted for: number of occupational risks (0, 1 or 2, 3 or 4, ≥5 risks), number of home-work conflicts (0, 1, 2, >2 risks), age group (<25, 25- <30, 30- < 35, ≥ 35 years), occupational group (managers/supervisors, intermediate occupations, employees + manual workers), type of contract (non-fixed term, fixed-term)

^d^Ajusted for: number of occupational risks (0, 1 or 2, 3 or 4, ≥5 risks)

^e^Among 17 selected occupational risks in this study

^f^Among preschool-age children at home (yes/no), home-work commuting > 50 min/d (yes/no), duration of working hours >8 h/d (yes/no), irregular working hours (yes/no), and absent of two consecutive rest days in a week (yes/no)

^g^Grouping manual workers and employees because of small number of manual workers

^h^Ajusted for: number of occupational risks (0, 1 or 2, 3 or 4, ≥5 risks), home-work commuting felt as difficult (yes/no), age group (<25, 25- <30, 30- < 35, ≥ 35 years), occupational group (managers/supervisors, intermediate occupations, employees + manual workers), company size (<10, 10–49, 50–199, ≥ 200 workers), type of contract (non-fixed term, fixed-term)

^i^Ajusted for: number of occupational risks (0, 1 or 2, 3 or 4, ≥5 risks), number of home-work conflicts (0, 1, 2, >2 risks)

Table 3 shows the RRa for “early SL” (<15 WG) without returning to work until delivery (Table 2). The variables “number of occupational hazards”, deprivation and “pregnancy at risk” were found to be associated with increased RRa of SL compared with the reference group. We always observed a gradient with “number of occupational hazards”. Overall, 14.4% of pregnant workers left their job before 15 WG.

**Table 3:** Bernoulli generalized linear model with “early sick leave” (SL) without returning to work until delivery (<15 WG) as dependent variable

	Early sick leave (*N* = 1257)		
	*RRa* ^a^	*95% CI*	*p*
Number of occupational risks^b^			
No risk	Ref	-	*<0.001*
1 or 2 risks	1.48	0.92 - 2.38	
3 or 4 risks	2.03	1.25 - 3.30	
≥ 5 risks	2.90	1.89 - 4.44	
Deprivation (EPICES score ≥ 30)			
No	Ref	-	*0.005*
Yes	1.45	1.12 - 1.88	
Pregnancy at risk			
No	Ref		*<0.001*
Yes	2.42	1.87 - 3.12	

*RRa* risk, *95% CI* 95% confidence intervals, *p* p-value, *WG* week gestation

^a^Ajusted for: number of occupational risks (0, 1 or 2, 3 or 4, ≥ 5 risks), deprivation, EPICES score ≥ 30” (yes/no), pregnancy at risk (yes/no)

^b^Among 17 selected occupational risks in this study

Table 4 displays the results for the ZINB model (Table 4). The logistic portion is based on the probability of zero days of SL. This probability decreased with the number of “occupational hazards for pregnancy”, “work-home conflicts” or for “pregnancy at risk”. Working in a small company or having a non-fixed term contract was also associated with no SL. For the negative binomial portion, the duration of SL was found to be clearly associated with “number of occupational hazards”, “work-family conflicts”, deprivation and “pregnancy at risk”. Here also the gradient with “number of occupational hazards” was reported.

**Table 4:** Zero-inflated negative binomial model for number days of sick leave before pathological and legal maternity leaves

	Logistic portion^a^				Negative binomial portion				
	β^b^	95% CI	*p*	Probability of being an extra zero	β^b^	95% CI	*p*	Adjusted mean NOLD^c^	Difference (factor-intercept)^c^
Intercept	0.92	−0.08 – 1.91	*0.07*	0.71	3.46	3.06 – 3.87	*<0.001*	31.9	0
Number of occupational risks									
1 or 2 risks *vs* 0 risk	−0.44	−0.83 – -0.06	*<0.001*	0.62	0.19	0.03 – 0.35	*<0.001*	38.6	+06.7
3 or 4 risks *vs* 0 risk	−0.74	−1.23 – -0.24		0.54	0.38	0.19 – 0.57		46.8	+14.9
≥ 5 risks *vs* 0 risk	−1.05	−1.54 – -0.57		0.46	0.52	0.35 – 0.70		53.8	+21.9
Work-family conflict									
1 risks *vs* 0 risk	0.14	−0.23 – 0.52	*0.35*	0.74	0.20	0.04 – 0.35	*0.03*	38.8	+6.9
2 risks *vs* 0 risk	−0.14	−0.60 – 0.32		0.68	0.23	0.06 – 0.39		40.0	+8.1
≥ 3 risks *vs* 0 risk	−0.33	−0.99 – 0.34		0.64	0.21	0.01 – 0.41		39.4	+7.6
Deprivation (EPICES score ≥ 30)									
Yes *vs* no	−0.17	−0.59 – 0.25	*0.42*	0.68	0.17	0.04 – 0.31	*0.01*	37.9	+6.0
Working time									
Full-time *vs* part-time	0.15	−0.24 – 0.53	*0.46*	0.74	−0.15	−0.29 – -0.01	*0.03*	27.4	−4.5
Home-work commuting felt as difficult									
Yes vs no			*<0.001*	0.53	0.09	−0.04 – -0.22	*0.17*	35.0	+3.1
Pregnancy at risk									
Yes *vs* no	−1.26	−1.72 – -0.80	*<0.001*	0.41	0.42	0.29 – 0.54	*<0.001*	48.3	+16.5
Company size (workers)									
≥ 50 -199 *vs* ≥ 200	0.64	0.09 – 1.18	*0.02*	0.68	0.07	−0.12 – 0.25	*0.34*	34.0	+2.1
10 – 49 *vs* ≥ 200	0.69	0.17 – 1.20		0.83	0.02	−0.14 – 0.19		32.7	+0.8
< 10 *vs* ≥ 200	0.29	−0.25 – 0.82		0.77	−0.08	−0.24 – 0.09		29.4	−2.4
Type of contract									
Fixed-term vs non-fixed term	−1.03	−1,71 – -0.35	*0.003*	0.47	0.26	−0.06 – 0.58	*0.11*	41.2	+9.4

*RRa* ajusted relative risk, *95% CI* 95% confidence intervals, *p* p-value, *NOLD* number of sick leave days vs: versus

^a^Modeling the probability of being an extra zero

^b^Ajusted for: number of occupational risks (0, 1 or 2, 3 or 4, ≥5 risks), number of home-work conflicts (0, 1, 2, ≥3 risks), deprivation EPICES score ≥ 30 (yes/no), pregnancy at risk (yes/no), working time (full-time, part-time), company size (<10, 10–49, ≥ 50 – 199,≥ 200 workers), type of contract (non-fixed term, fixed term), home-work commuting felt as difficult (yes/no)

^c^in days

## Discussion

In our study, the rate of SL in pregnant workers was very high. After adjustment, the cumulative index of occupational hazards for pregnancy was clearly associated with SL (for “at least one leave”, according to duration and for “early leave”) in a dose–response relationship. We also found deprivation, pregnancy at risk, ART, work-family conflicts, home-work commuting felt as difficult, and young age to be associated with a higher risk of SL. For some factors, the results varied according to the trimesters of pregnancy. Moreover, we observed that there were less SL in small companies or for non-fixed term contract to 2nd or 3rd trimester.

The study has a number of strengths and limitations. Among its strengths, it is noteworthy that exposure data and outcomes were collected independently of each other. The assessment of occupational exposures was carried out by OHPs with good knowledge of the workstations and the workers answered the questionnaire before visiting the OHPs. Also our results were improved by adjustment for socioeconomic deprivation based on the EPICES scale [12, 31]. The other advantages of our study were its large size and the wide range of possible confounders collected, for which we adjusted our data. The large participation of OHPs in two French regions throughout the duration of the study and the high participation rate also improved the generalisability of our work. However, several potential limitations exist. High absence rates among pregnant workers may be caused by other characteristics not captured by the variables chosen in our study (e.g. job strain, other psychosocial factors) [19]. Information on outcomes was based on retrospectively collected self-reported information which may lead to errors with respect to both time of occurrence and duration. However, according to some studies, the duration from self-reported data tend to be shorter than those based on recorded data and agreement between self-reported and recorded days of absence decreased as the total number of days increased [32, 33]. Consequently, our results tend to rather underestimate reality. Also we did not have access to medical records relating to maternity and information on “pregnancy at risk” was based on self-reporting by workers. There was no consensual definition and literature data on this variable are scarce [34, 35]. In a report from the French National Authority for Health, the rate of “pregnancy at risk” was 20% versus 24.8% for our study [34]. Our rate was probably overestimated since there was no medical control of the declarations from the workers.

The high rate of “at least one SL” (74.9%) in our study is similar to that of a Norwegian study (75.3%, similar definition) [20] but higher than other studies from Norway (51%) [24] and from Denmark (31%) [7]. Our rate of “early leave” (14.4% before 15 WG) was consistent with the results from other studies: 27.5% before 24 WG [15]; 43% before 28 WG [36]. With respect to the average number of days’ absence without inclusion of the 15 days accepted for pathological leave, one study in French hospitals from 2005 to 2008 using data obtained from employers’ records reported 33.6 days on average versus 31.9 to 53.8 days for our study (see Table 3) [9]. But it is difficult to compare the different studies globally because the populations studied and the definitions chosen are not similar [4, 19].

SL is expected when a pregnant woman is sick or medically at risk as this was confirmed by our results with the variables “pregnancy at risk” and ART, and reported by other studies [12, 15, 20]. However, our results adjusted for “pregnancy at risk” and ART argue strongly for the independent implication of occupational factors in the decision to take SL. Several studies have also reported that some working conditions were statistically related to SL: heavy lifting [7, 19, 37]; work with a lot of walking or standing [7]; uncomfortable working positions [7, 19]; night or shift work [7, 19, 24]; long working days [7]. Several studies have reported an association between the combination of occupational hazards and SL [9, 18, 37]. A French study in 1985 on hospital workers reported a longer duration of SL (including the supplementary week for pathological leave) was associated with the accumulation of occupational factors: 37 days on average for 0 to 1 risk; 50 days on average for 2 or 3 risks [18]. In another French study on hospital workers in 2008, the average number of days’ absence after exclusion of women having had a pregnancy-related illness, increased with the physical load of the workstations [9].

Like other authors, we reported an association between early SL and socioeconomic deprivation in pregnant workers [12, 20]. From literature data, early SL was more frequent among pregnant workers with unstable jobs and with less-qualified occupational categories [12]. Deprived pregnant workers were exposed to more occupational hazards for pregnancy and higher risk of pregnancy-related illnesses [12, 31]. In France, pregnant workers can adapt their jobs using occupational health services, but not all women seem to benefit from those adjustments. Consequently, some deprived pregnant workers could leave early the workplace for those different reasons. In France in 2014 before legal or pathological leaves, except for some great companies, ordinary sick leaves were paid to 50% of the salary. From our study, pregnant workers with social vulnerabilities may decide to leave their job earlier than they legally can, even if they lose part of their income [12]. But this could have financial and social important consequences (isolation, less medical care). It is important to note that, in contrast with our result about early SL, sick leaves paid to 50% of the salary could also affect the use of sick leave among the poorest workers frequently exposed to occupational hazards during pregnancy (particularly of a physical nature) and could have some consequences on adverse perinatal outcomes [31]. One French study has reported that deprived pregnant workers exposed to three or more occupational hazards were significantly associated with pre-term birth [31].

We observed an association between “at least one SL” and “number of home-work conflicts” or “home-work commuting felt as difficult”. Fatigue, pain or discomfort during pregnancy are expected to be more frequent in the last trimesters than in the 1st trimester and could explain these specific associations. However, some women also still have the double burden of combining their household and child-rearing responsibilities with their jobs [11]. Pregnant workers may need to leave their job when this double burden can no longer be ensured [11]. Furthermore, we found an association between “at least one SL” and younger women (<25 years), as in certain other studies [8, 11, 16], but not others with different cut-offs [7, 19]. First pregnancy and inexperience related to age or specific representation such as “absence from work is considered beneficial for the child” may explain this result [11]. In our study, the relationship between occupational classes and SL after adjustment is not clear. This may be explained by a lower SL rate in the reference group rather than higher rates in other groups; underreporting of SL cannot be ruled out, because in general managers/supervisors are averse to declaring SL. In contrast, the observations of less SL in small companies or for non-fixed term contract are expected results because the risk losing his job (during or after pregnancy) is more important in these situations.

Our study has some practical perspectives. French labor law provides for a visit with an OHP after delivery but not before [3]. Consequently, few pregnant workers see an OHP during pregnancy. However, the situation for an OHP who receives pregnant workers may be difficult if they cannot propose job adjustments or other preventive measures in the workplace. Indeed, sometimes job adjustment is not possible because some employers may prefer pregnant workers to take sick leave so they can employ a healthy person to replace them during their absence [37]. Moreover, social conditions and, specific attitudes (for example, related to health beliefs) among pregnant workers may explain some SLs [16]. Also, some prenatal caregivers who care for pregnant workers without real knowledge of the workstation and without contact with OHPs may opt to prescribe SL to prevent disorders associated with pregnancy [21, 22]. At present, the management of employment during pregnancy results from complex interplay between the worker, employer, prenatal caregiver and, other influences [21, 22]. In addition, some pregnant workers are likely excluded from work without having had an assessment of possible preventive measures in the workplace [2, 31]. When occupational hazards for pregnancy exist, our results argue for a risk assessment in the workplace during early pregnancy, preferably with an OHP [3]. The aim would be to avoid reproductive risks by removing hazards or adjusting work [2, 3, 23].

## Conclusion

Our results argue for the implication of exposure of the occupational hazards for pregnancy as an explanation for the occurrence of SL. More prevention in the workplace for pregnant workers exposed to occupational hazards could reduce SL. Further studies are needed to demonstrate that reducing SL might not be associated with a higher number of pregnancy-related illnesses and convince all the stakeholders (workers, employers, caregivers) to go in this direction. Our results also suggest that the duration and occurrence of early SL are significant in deprived pregnant workers. In addition, future studies should also take into account this vulnerable population in their analyses.

## Abbreviations

ART: Assisted reproductive therapy
OHPs: Occupational health physicians
RRa: Adjusted relative risks
SL: Sick leave
WG: Week of gestation
ZINB: Zero-inflated negative binomial
